# Reemergence of Canine *Echinococcus granulosus* Infection, Wales

**DOI:** 10.3201/eid1104.040178

**Published:** 2005-04

**Authors:** Imad Buishi, Tom Walters, Zoë Guildea, Philip Craig, Stephen Palmer

**Affiliations:** * University of Salford, Greater Manchester, United Kingdom;; † Powys Health Promotion Unit, Powys, Wales, United Kingdom;; ‡ University of Wales, Cardiff, Wales, United Kingdom

**Keywords:** Echinococcus granulosus, Epidemiology, Risk factors, Coproantigen ELISA, Powys, FMD, Research, Prevalence

## Abstract

As a consequence of large-scale outdoor slaughter of sheep during the 2001 foot and mouth disease (FMD) outbreak in the United Kingdom and the possibility of increased risk for transmission of *Echinococcus granulosus* between sheep and dogs, a large survey of canine echinococcosis was undertaken in mid-Wales in 2002. An *Echinococcus* coproantigen-positive rate of 8.1% (94/1,164) was recorded on 22% of farms surveyed, which compares to a rate of 3.4% obtained in the same region in 1993. Positivity rates between FMD-affected properties and unaffected ones did not differ significantly. Significant risk factors for positive results in farm dogs were allowing dogs to roam free and the infrequent dosing (>4-month intervals) of dogs with praziquantel. When these data are compared to those of a previous pilot hydatid control program in the area (1983–1989), an increase in transmission to humans appears probable.

## Abstract 2

A survey of 2 Welsh counties found that canine echinococcosis has reemerged.

*Echinococcus granulosus* infection in sheep and dogs has been known to be endemic in parts of Wales and the English borders for many decades (1–3). An analysis of national hospital records for the period 1974–1983 showed that the incidence of human cystic echinococcosis was 0.2 cases per million in England and 2 cases per million population in Wales, with highest rates (5.6 cases per million) occurring in southern Powys County (4). To reduce the incidence of human cystic echinococcosis (also called cystic hydatid disease), a voluntary hydatid control program of supervised dog dosings at 6 weekly intervals with praziquantel was introduced in south Powys in 1983 and continued until 1989 (5–7). Ovine hydatidosis rates in the intervention area dropped from 23.5% to 10.5% after that period, and experimental use of sentinel lambs confirmed that transmission of *E. granulosus* was significantly reduced by this regime (8). Trend analyses of hospital admissions of human hydatid disease showed that, by 1993, clinical cystic echinococcosis disease in children (<15 years old) had ceased in the intervention area. However, a new focus of human cystic echinococcosis was identified for the period 1984–1990 in an area bordering south Powys, namely, the northern parts of the counties of Gwent and mid-Glamorgan (7). Furthermore, canine echinococcosis rates, measured indirectly with an *Echinococcus*-specific coproantigen enzyme-linked immunosorbent assay (ELISA), reflected the clinical data for intervention and nonintervention areas (9).

In 1989, the supervised dog-dosing program was stopped and replaced by a health education program (T.M. Walters, pers. comm.). A follow-up abattoir and dog coproantigen survey in 1995 to 1996, however, indicated that *E. granulosus* infection had reemerged in sheep and dogs in the previous hydatid-control intervention areas (7). In 2001, the foot and mouth disease (FMD) epidemic in sheep in England and Wales (10) affected some farms in both the former hydatid-intervention and nonintervention areas. Concern was raised that dog access to carcasses of sheep, euthanized as part of the FMD control program and awaiting incineration, could amplify the prevalence of infection in dogs and thereby the subsequent risk for humans. A third coproantigen survey of farm dogs in south Powys and north Gwent was therefore undertaken in 2002 to determine the prevalence of canine echinococcosis in the former hydatid-intervention and nonintervention areas.

## Methods

### Design

The pre-FMD prevalence of canine echinococcosis was estimated to be 7% from previous surveys. We decided that a doubling of this value would represent a clinically important increase in prevalence. To detect an increase in prevalence from 7% in farms without FMD to 15% in farms with FMD, with a power of 80% and significance level of 5%, a sample of 150 dogs on FMD farms and 850 dogs on non-FMD farms was required.

Sampling took place during a 5-month period from July to November 2002. Farmers in the Welsh counties of Powys (previous hydatid-intervention area) and Gwent (nonintervention area) were randomly selected from a government listing of farms and invited by telephone to join the study. Farms were visited, dogs were sampled, and questionnaires were completed.

### Coproantigen Testing

Dogs were tethered or muzzled by owners, and a rectal fecal sample was taken from each dog with a plastic loop spatula. The specimen was mixed (1:1) with pre-prepared phosphate-buffered saline (pH 7.2) plus 0.3% Tween 20 (PBS-T) (11,12). Each sample was collected into a 5-mL screw-capped tube labeled with the dog identification and farm reference numbers. Fecal samples were initially stored frozen at –20°C for 5–10 weeks soon after collection, then transported to the pathogen laboratory at University of Salford and frozen at (–80°C) until tested. Before testing, fecal samples were thawed and mixed by hand shaking, then centrifuged at 500 x *g* for 10 min at room temperature. Fecal supernatants were tested for *Echinococcus* coproantigens by using a standard ELISA that used a capture antibody against *E. granulosus* adult somatic antigens (12) as had been used in the previous survey of canine echinococcosis in the Powys area (7).

### Risk Factors

Data were collected on previous history of human cases of cystic echinococcosis in the household, knowledge of hydatid disease, dog age and sex, frequency of dog anthelmintic treatment (by owner or veterinarian), nature of dog food, and how dogs were restrained. In addition, farmers were asked whether they had had FMD on their properties or if their livestock were slaughtered for condemnation or because of FMD contiguous culling. Dog owners were also asked if they slaughtered livestock even occasionally at or around their farm, and if so, where and how they disposed of slaughter offal.

The chi-square test was used to determine whether dog coproantigen prevalence differed significantly between the levels of selected possible risk factors. Odds ratios (ORs), p values, and 95% confidence intervals (CIs) were calculated to quantify the magnitude of these differences. Multilevel modeling was used to examine the effect of clustering of dogs within farms. Results showed that variation between farms was not significant. Consequently, a single-level multivariate logistic regression was carried out; all risk factors were entered as explanatory variables. Analyses were performed with SPSS (SPSS Inc., Chicago IL, USA) and MLWin (Institute of Education, London, UK).

## Results

In Powys, 473 farmers were eligible to take part; 72 (15%) did not have dogs, 112 (24%) could not be contacted, and 16 (3%) declined to cooperate. Therefore, a total of 273 farmers who were contacted and were eligible agreed to take part. Equivalent figures on response rates were unavailable for Gwent. A total of 1,178 dogs were on these farms (990 from Powys and 188 from Gwent), and fecal samples were obtained from 1,164 (588 dogs and 576 bitches). In 75 farms, sheep, cattle, or both had been slaughtered as part of FMD controls; FMD was recorded on 27 of these farms.

The *Echinococcus* coproantigen ELISA was positive in 94 (8.1%) of 1,164 of farm dogs. Of farms surveyed, 22% contained at least 1 dog with a positive coproantigen result. Prevalence of positive coproantigen tests in the previous hydatid-intervention area was 8.5% (79/928), compared with 6.4% (15/233) in the former nonintervention areas.

Univariate analysis of questionnaire data showed that male and female dog coproantigen-positive rates did not differ significantly. Younger dogs had a tendency for higher positive rates (≤5 years, p = 0.03). Prevalence of dog coproantigen positivity was significantly associated with farm onsite slaughter of sheep (home-slaughter), the occurrence of free-roaming dogs, and low frequency of dog anthelmintic treatment by owners (Table 1). Coproantigen-positive rates in dogs for FMD and non-FMD farms were similar. Farms that reported feeding food scraps or offal to dogs, or allowing them to scavenge freely, were also associated with a significantly higher risk of an *Echinococcus* coproantigen-positive result (Table 1).

**Table 1:** Univariate analysis of *Echinococcus* coproantigen ELISA data from Welsh farm dogs and possible risk factors determined by questionnaire*

Risk factor	No. positive/negative for coproantigen† (N = 1,164)				
	Present	Absent	OR	95% CI	p value
Farmer slaughters sheep	11/54	83/1,016	2.49	1.26–4.95	0.007
Stock euthanized because of FMD	20/255	74/815	0.86	0.52–1.44	0.576
FMD-affected farm	8/89	85/975	1.03	0.48–2.2	0.937
Dog free roaming	69/504	22/519‡	3.23	1.97–5.3	<0.0001
No disease knowledge	24/230	70/840	1.25	0.77–2.04	0.364
Dosing interval every 4–6 mo	63/554	18/434§	2.7	1.6–4.7	<0.0001
Dosing interval >6 mo	13/81	18/434§	3.87	1.83–8.21	<0.0001
Dog sex (male)	43/543	50/526	0.83	0.55–1.27	0.4
Age of dog ≤5 y	63/602	30/467	1.63	1.04–2.56	0.033
Food: scraps or offal	18/122	76/947¶	1.84	1.06–3.18	0.027

*Sampled in Powys and Gwent, Wales, United Kingdom, July–November 2002. ELISA, enzyme-linked immunosorbent assay; OR, odds ratio; CI, confidence interval; FMD, foot and mouth disease. †By enzyme-linked immunosorbent assay. ‡Dog chained up. §Dosing <4 months. ¶Boiled food.

Multilevel modeling was used to examine risk factors for dogs, farms, and districts, but the variation between farms was not significant. In a single-level multivariate logistic regression model that used backward stepwise variable selection, allowing a dog to roam free and administering anthelmintic treatment infrequently were significant risk factors for *Echinococcus* coproantigen positivity (Table 2). Using this logistic regression model, we calculated, for example, that a dog that had been reportedly prevented from roaming free and that had received anthelmintic treatment 4 times a year had a probability of a positive coproantigen result of 0.07 {1/[1+ exp –(-2.52)]}. In contrast, a dog that was allowed to roam and was given anthelmintic treatment only once per year had a probability of 0.4 {1/ [1+exp – (-2.52+1.15+1.07)]} for an *Echinococcus*-positive coproantigen result.

**Table 2:** Multivariate logistic regression model of possible risk factors for a positive Echinococcus coproantigen-test result in farm dogs (n = 1,164)

Risk factor	p value	Regression coefficient	OR	95% CI
Location of dog (compared to chained dogs)				
Free roaming	<0.0001	1.07	2.91	1.77–4.8
Other	0.411	0.53	1.7	0.48–5.91
Frequency of anthelmintic treatment (compared to <4 months)				
4–6 months	0.002	0.84	2.31	1.35–3.95
>6 months	0.004	1.15	3.16	1.46–6.85
Constant	<0.0001	–2.52		

*Sampled July–November 2002, Powys and Gwent Counties, Wales. OR, odds ratio; CI, confidence interval.

## Discussion

We conducted a study to investigate the concern that human cystic hydatid disease, or cystic echinococcosis, could reemerge in mid-Wales as a consequence of FMD control measures in which large numbers of sheep carcasses lying in fields were potentially accessible to dogs and foxes for several weeks. Although we found no statistical association with FMD-affected farms, we did confirm that canine echinococcosis had reemerged in dogs living in previously hydatid-free areas. The coproantigen-positive rate of 8% in the 2002 farm dog survey was significantly higher than the 3.4% prevalence recorded in the same localities in 1989 to 1993 (7). Risk factors for coproantigen-positive dogs were allowing farm dogs to roam free and infrequent anthelmintic treatment.

Why an association between canine echinococcosis coproantigen positivity and FMD-affected properties was not found is not clear. The lack of an association may be because the large piles of FMD-culled sheep carcasses were in designated government-regulated areas with relatively poor access for scavenging dogs. Moreover, dogs from non-FMD farms might have had the same access to culled carcasses as dogs from FMD farms. However, we were not able to identify whether dogs from non–FMD-affected farms had access to carcasses of slaughtered sheep on FMD farms. Dogs that scavenged may also have been already infected.

The absence of a statistical association with FMD and the findings of earlier surveys suggest that the high prevalence of canine echinococcosis is due to failure of the control strategy. The supervised dog-dosing hydatid-intervention program initiated in south Powys under the auspices of the U.K. Ministry of Agriculture, Fisheries and Food from 1983 to 1989 reduced transmission of *E. granulosus* to sentinel lambs. Moreover, the incidence of hospital-treated human cystic echinococcosis disease in the intervention area in Powys fell from 4/100,000 to 2.3/100,000 from 1984 to 1990. In May 1993, prevalence of *Echinococcus* in dogs, as measured by a highly genus-specific coproantigen test, was 0% in the intervention area (lower 95% CI 0%–3.4%). However, 10%–18% of older sheep (>3 years) at slaughter remained infected (7).

In 1989, the supervised dog-dosing program was replaced by a health education program aimed at schoolchildren and farmers in Powys to encourage dog owners to dose their dogs every 6 weeks. Following this, a sentinel lamb study in the former intervention area from 1995 to 1996 (13) found that 5% of sentinel lambs became infected (10% in the adjacent nonintervention areas), indicating that pastures were recontaminated. A coproantigen survey of farm dogs at the same time (1995–1996) showed that 6% of dogs in the former intervention area were positive as were 24% of those in nonintervention areas (13). Our study used the same *Echinococcus*-specific coproantigen ELISA (11,12) and indicated that *E. granulosus* tapeworm infection in dogs was widespread in both the former intervention and nonintervention areas. Because of the latency of human cystic echinococcosis, children and adults exposed to infected dogs since 1989 may not have clinical disease for several decades.

A study conducted to evaluate the effectiveness of the health educational program on control of transmission of *E. granulosus* in south Powys (after 1989) demonstrated that the educational campaign alone was unable or insufficient to prevent transmission of *E. granulosus* in that region. This study also suggested that lifting the "fast-track" attack phase of treating dogs every 6 weeks with praziquantel after only 5 years was premature (13).

Reemergence of human cystic echinococcosis as a public health problem has occurred in other countries when hydatid-control programs ceased. For example, in Cyprus, cystic echinococcosis reemerged after the breakdown of an islandwide hydatid-control program following partitioning of the island (14). In Bulgaria, human cystic echinococcosis rates increased after hydatid campaigns were ended or reduced (15). And in Kazakhstan, human cystic echinococcosis incidence rates increased 4-fold within 10 years after post-Soviet independence, with its dismantling of collectives and changes in organized livestock and farming practices (16).

In conclusion, reemergence of *E. granulosus* in dogs in the last 10 years in south Powys, Wales, appears to be due to the withdrawal of the supervised dog-dosing control scheme and a reversion to risky practices of farmers (e.g., allowing farm dogs to roam free and infrequent or no anthelmintic treatment). The FMD outbreak in 2001 did not appear to increase the risk for canine echinococcosis. Urgent efforts are needed to address farm dog owner–associated risk factors if hydatid disease is to be brought back under control in this area.
